# Whole-Transcriptome RNA Sequencing Uncovers the Global Expression Changes and RNA Regulatory Networks in Duck Embryonic Myogenesis

**DOI:** 10.3390/ijms242216387

**Published:** 2023-11-16

**Authors:** Shuibing Liu, Jintao Wu, Wentao Zhang, Hongxia Jiang, Yanan Zhou, Jing Liu, Huirong Mao, Sanfeng Liu, Biao Chen

**Affiliations:** 1 College of Animal Science and Technology, Jiangxi Agricultural University, Nanchang 330045, China; 18879138275@163.com (S.L.); 18879129396@163.com (J.W.); zwt0821@163.com (W.Z.); jhx13340646380@163.com (H.J.); qianshishizhimao@163.com (Y.Z.); jingliuexon@163.com (J.L.); huirongm82@126.com (H.M.); 2 Poultry Research Institute, Jiangxi Agricultural University, Nanchang 330045, China

**Keywords:** duck myogenesis, whole-transcriptome sequencing, ceRNA regulatory network, non-coding RNAs

## Abstract

Duck meat is pivotal in providing high-quality protein for human nutrition, underscoring the importance of studying duck myogenesis. The regulatory mechanisms governing duck myogenesis involve both coding and non-coding RNAs, yet their specific expression patterns and molecular mechanisms remain elusive. To address this knowledge gap, we performed expression profiling analyses of mRNAs, lncRNAs, circRNAs, and miRNAs involved in duck myogenesis using whole-transcriptome RNA-seq. Our analysis identified 1733 differentially expressed (DE)-mRNAs, 1116 DE-lncRNAs, 54 DE-circRNAs, and 174 DE-miRNAs when comparing myoblasts and myotubes. A GO analysis highlighted the enrichment of DE molecules in the extracellular region, protein binding, and exocyst. A KEGG analysis pinpointed pathways related to ferroptosis, PPAR signaling, nitrogen metabolism, cell cycle, cardiac muscle contraction, glycerolipid metabolism, and actin cytoskeleton. A total of 51 trans-acting lncRNAs, including *ENSAPLT00020002101* and *ENSAPLT00020012069*, were predicted to participate in regulating myoblast proliferation and differentiation. Based on the ceRNAs, we constructed lncRNA-miRNA-mRNA and circRNA-miRNA-mRNA ceRNA networks involving five miRNAs (miR-129-5p, miR-133a-5p, miR-22-3p, miR-27b-3p, and let-7b-5p) that are relevant to myogenesis. Furthermore, the GO and KEGG analyses of the DE-mRNAs within the ceRNA network underscored the significant enrichment of the glycerolipid metabolism pathway. We identified five different DE-mRNAs, specifically *ENSAPLG00020001677*, *ENSAPLG00020002183*, *ENSAPLG00020005019*, *ENSAPLG00020010497*, and *ENSAPLG00020017682*, as potential target genes that are crucial for myogenesis in the context of glycerolipid metabolism. These five mRNAs are integral to ceRNA networks, with miR-107_R-2 and miR-1260 emerging as key regulators. In summary, this study provides a valuable resource elucidating the intricate interplay of mRNA-lncRNA-circRNA-miRNA in duck myogenesis, shedding light on the molecular mechanisms that govern this critical biological process.

## 1. Introduction

China is the world’s foremost producer of duck meat [1], which is recognized for its exceptional protein content and nutritional value [2]. Skeletal muscle, a crucial component of poultry, accounts for 50% of chicken weight, exerting a pivotal influence on metabolic processes [3]. The feed conversion rate and meat yield are important economic indicators in animal husbandry, and the development of skeletal muscle is closely linked to the meat production in livestock. Therefore, it is of great significance to explore the molecular mechanism linked to the skeletal muscle development of ducks.

At the embryonic stage, myoblasts play pivotal roles in shaping skeletal muscle. They orchestrate complex biological processes, including migration, adhesion, proliferation, membrane recombination, and nuclear fusion, culminating in the formation of multinucleated myotubes [4]. In birds, the initiation of muscle tissue commences during the embryonic phase, with muscle cells expanding or undergoing hypertrophy post-hatching through satellite cell fusion [5,6]. 

Genes, as the repositories of genetic information, govern an array of life’s biological processes. While gene products, including polypeptides and protein macromolecules, play vital biological functions [7], a mere 2% of the human genome comprises protein-encoding genes. The majority of genome sites, over 98%, are transcribed into non-coding RNAs (ncRNAs) [8]. An expanding body of research has shown that ncRNAs such as lncRNA [9], circRNA [10], and microRNA [11] can form a complex post-transcriptional regulatory network to regulate gene expression and affect the growth and development of skeletal muscle. 

Until now, investigations into ncRNAs linked to duck skeletal muscle growth and development have predominantly focused on characterizing [12,13] or exploring the function of individual RNA types [14,15]. While previous studies have successfully revealed competing endogenous RNA (ceRNA) regulatory networks governing aspects like chicken fat deposition and skeletal muscle growth and development [16,17], a comprehensive ceRNA regulatory network, comprising lncRNA/circRNA-miRNA-mRNA interactions during duck embryonic myoblast proliferation and differentiation, remains unknown. 

**Figure 1:**
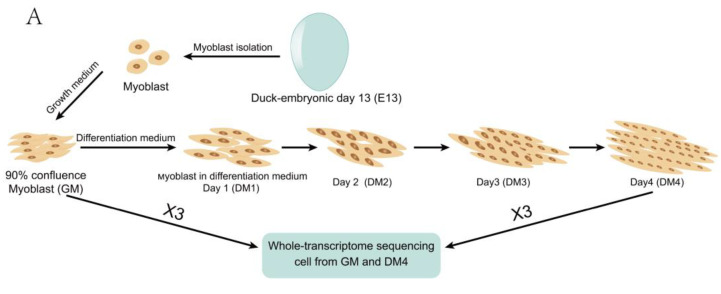

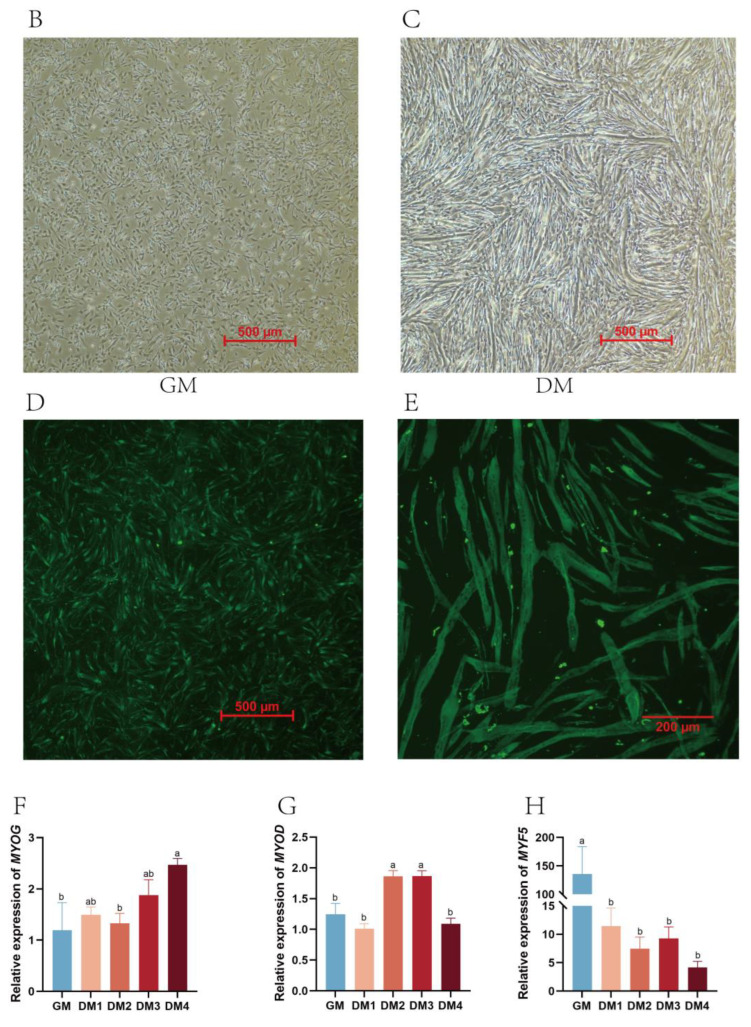
Duck primary embryonic myogenesis. (**A**) Flow chart of the experimental procedure. (**B**) Proliferating myoblasts. (**C**) Differentiating myoblasts on day 4 (myotubes). (**D**) Immunofluorescence analysis of undifferentiated duck myoblasts was maintained in GM and stained with anti-desmin. (**E**) Immunofluorescence analysis of differentiating myoblasts on day 4 stained with anti-MyHC. Relative expression of muscle markers, including *MYOG* (**F**), *MYOD* (**G**), and *MYF5* (**H**), during in vitro differentiation of duck primary myoblasts. RNA was isolated on days 0 (GM), 1 (DM1), 2 (DM2), 3 (DM3), and 4 (DM4) of myoblast differentiation culture. Data (4 biological replicates) are presented as means ± s.e.m. Different letters between two groups represent significant differences (*p* < 0.05).

This study contributes whole-transcriptome RNA sequencing and global expression data (mRNA-lncRNA-circRNA-miRNA) on duck embryonic leg muscle, including myoblasts in a growth medium and differentiating myoblasts (myotubes) on day 4. We present a predicted a ceRNA regulatory network for duck embryonic myogenesis, elucidated in Figure 1A. These data substantially enhance our understanding of the fundamental molecular mechanisms underlying duck myogenesis and serve as a valuable reference for future myogenesis studies.

## 2. Results

### 2.1. Duck Primary Myoblast Differentiation 

Duck primary myoblasts were isolated from 13-day-old embryo leg muscle and cultured in a growth medium (GM) (Figure 1B). After inducing differentiation, myotube growth and enlargement were evident on day 4, as depicted in Figure 1C. Undifferentiated duck myoblasts were identified using a primary antibody against desmin and maintained in GM (Figure 1D). The cells that were exposed to a differentiation medium for four days (Figure 1E) were stained with a primary antibody against MyHC and a green fluorescein-conjugated secondary antibody, demonstrating the formation of myotubes on day 4, a contrast to the absence of myotubes during the GM culture. Additionally, the duck myoblasts were differentiated for four days (DM1 to DM4) to assess the mRNA levels of well-known muscle markers, including *MYOG*, *MYOD*, and *MYF5*, using qRT-PCR (Figure 1F–H). These findings affirm the induction of duck myogenesis, serving as a foundation for a subsequent analysis.

### 2.2. Expression Patterns of mRNAs, lncRNAs, and circRNAs in Duck Myogenesis

The total RNA was extracted from three replicates of duck myoblasts (GM1, GM2, and GM3) and myotubes (DM1, DM2, and DM3). Subsequently, RNA-seq was conducted, and the computational pipelines, detailed in Materials and Methods, were employed to identify mRNAs, lncRNAs, and circRNAs. Each cDNA library yielded over 61 million raw sequencing reads. Following meticulous filtering, the alignment rates to the Mallard duck reference genome exceeded 89.27% for all samples (Supplementary Table S1). A total of 14,028 mRNAs, 869 known lncRNAs, 5471 novel lncRNAs, and 7179 circRNAs were identified in myoblasts and myotubes (Supplementary Table S2).

### 2.3. Characteristics of Duck lncRNAs and mRNAs

**Figure 2:**
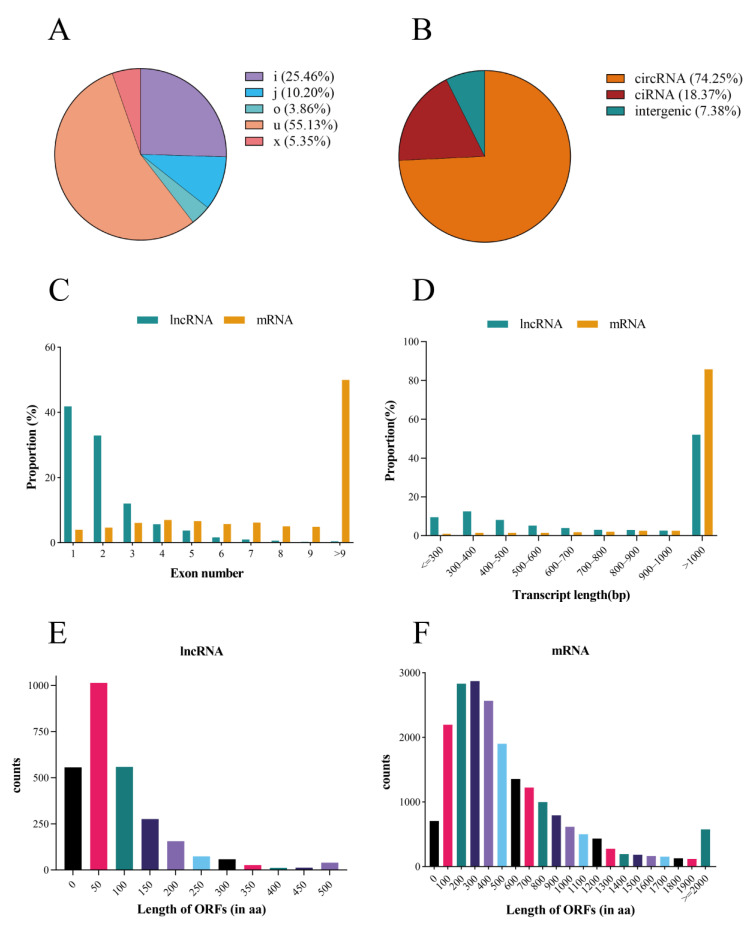
Features of duck lncRNA, mRNA, and circRNA. (**A**) The positional classification and proportion of lncRNAs. A transcript with one of the class codes, ‘i, j, o, u, and x’, was defined as a lncRNA transcript. (i) Class ‘i’ refers to a transcribed fragment, which could be either in the sense or anti-sense orientation, that is entirely contained within a reference intron. (j) Class ‘j’ pertains to transcripts that exhibit at least one splicing junction shared with the reference transcript. (u) Class ‘u’ designates a lncRNA as an intergenic transcript of unknown function. (o) Class ‘o’ is applied when an ordinary exon of a predicted lncRNA partially overlaps with a reference transcript. (**B**) The type and proportion of circRNAs. (circRNA) exon-derived circular RNA. (ciRNA) intron-derived circular RNA. (intergenic) intergenic-derived circular RNA. (**C**) Distribution of exon numbers for lncRNA and mRNA. (**D**) Transcript lengths of lncRNA and mRNA. Lengths of ORFs for lncRNA (**E**) and mRNA (**F**). nt: nucleotides; aa: amino acids.

The duck lncRNAs were classified into five types, with the majority being ‘u’ (55.13%), and a smaller fraction categorized as ‘x’ (5.35%). Meanwhile, 74.25% of the identified circRNAs originated from classical exons (Figure 2A,B). Considerable distinctions emerged in the transcript length, exon count, and open reading frame (ORF) between lncRNAs and mRNAs. Specifically, 86.69% of the lncRNAs featured 1–3 exons, while 49.98% of the mRNAs had 9 exons (Figure 2C). Moreover, 51.99% of the lncRNAs exceeded 1000 nucleotides in length, in contrast to 85.76% of the mRNAs (Figure 2D). In terms of the ORF length, 76.45% of the lncRNAs exhibited ORFs spanning 0–150 amino acids, whereas 59.5% of the mRNAs featured ORFs extending 100–600 amino acids (Figure 2E,F).

### 2.4. Differential Expression and Enrichment Analysis of mRNAs, lncRNAs, and circRNAs

**Figure 3:**
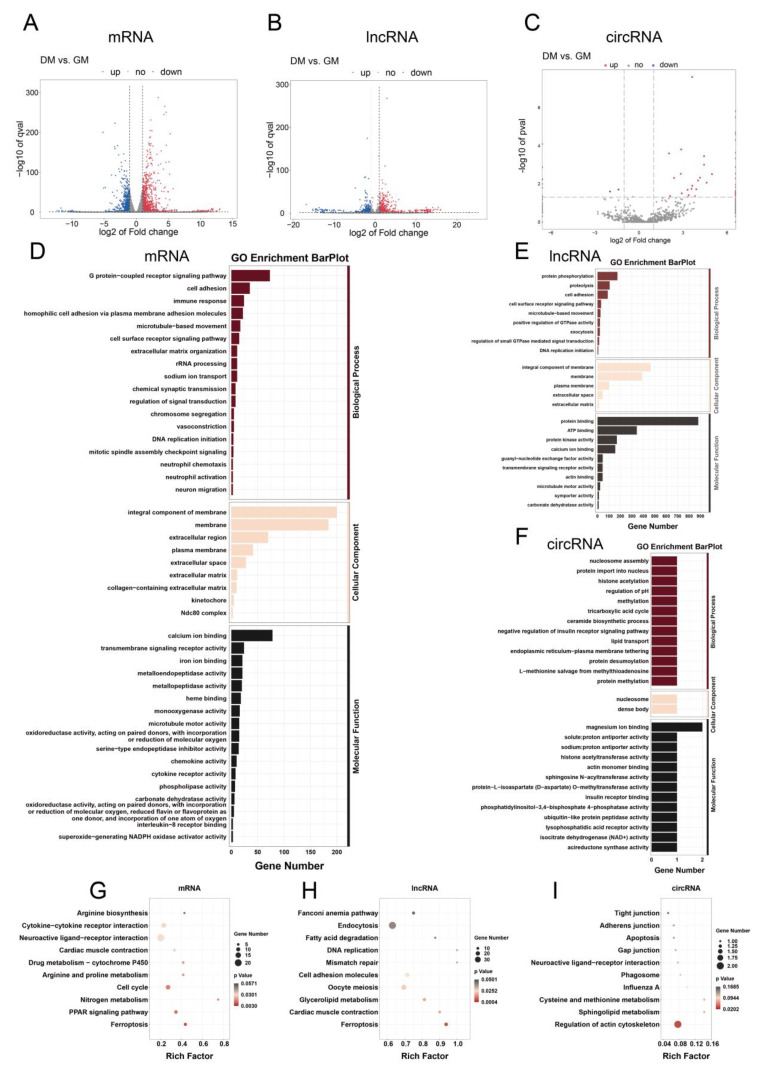
Differential expression analysis of mRNAs, circRNAs, and lncRNAs during duck primary myoblast differentiation. Volcano plots of gene expression levels of all mRNAs (**A**), lncRNAs (**B**), and circRNAs (**C**). The vertical dotted lines indicate |log2FC| = 1, and the horizontal dotted lines indicate *p* value = 0.05. GO (**D**) and KEGG (**G**) analyses of DE-mRNAs. GO (**E**) and KEGG (**H**) analyses of DE-lncRNAs. GO (**F**) and KEGG (**I**) analyses of DE-circRNAs.

The differential expression and distribution of mRNAs, lncRNAs, and circRNAs are visually represented in volcano plots (Figure 3A–C). Heatmaps further illustrate the expression differences between myoblasts and myotubes for mRNAs, lncRNAs, and circRNAs (Figure S1A–C). A comprehensive analysis between myoblasts and myotubes identified 1733 DE-mRNAs (1065 up-regulated and 668 down-regulated), 1116 DE-lncRNAs (653 up-regulated and 463 down-regulated), and 54 DE-circRNAs (44 up-regulated and 10 down-regulated) (Table 1 and Supplementary Tables S2–S5). A functional enrichment analysis of DE-mRNAs in duck myogenesis encompassed the GO analyses and KEGG pathway analysis. The DE-mRNAs between myoblasts and myotubes were notably enriched in 116 GO terms, with the top terms related to homophilic cell adhesion via plasma membrane adhesion molecules, extracellular matrix organization, rRNA processing, and microtubule-based movement (Figure 3D and Supplementary Table S3). The KEGG pathway analysis demonstrated enrichment in pathways including ferroptosis, PPAR signaling, nitrogen metabolism, and the cell cycle (Figure 3G and Supplementary Table S3). The DE-lncRNAs displayed significant enrichment in 74 GO terms, with prominent terms, such as cell adhesion, protein phosphorylation, proteolysis cell surface receptor signaling pathway, and the positive regulation of the GTPase activity (Figure 3E and Supplementary Table S4). The KEGG analysis revealed enrichment in pathways including ferroptosis, cardiac muscle contraction, and glycerolipid metabolism (Figure 3H and Supplementary Table S4). The host genes for DE-circRNAs exhibited significant enrichment in the GO terms associated with lipid transport, endoplasmic reticulum–plasma membrane tethering, and protein desumoylation (Figure 3F and Supplementary Table S5), while the KEGG analysis highlighted the regulation of the actin cytoskeleton (Figure 3I and Supplementary Table S5).

### 2.5. Analysis of the Interaction between Trans-Acting lncRNAs and mRNAs

**Figure 4:**
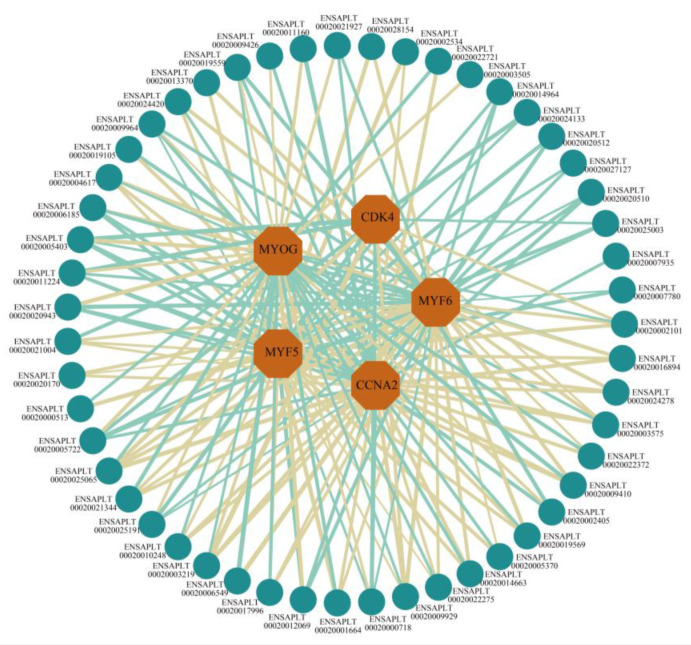
A regulatory network of trans-acting DE-lncRNAs and DE-mRNAs. Teal circles represent lncRNAs, and orange octagons represent mRNAs. The thickness of lines indicates the magnitude of free energy; the smaller lines indicate less free energy, and the thicker lines indicate more free energy. Light yellow lines indicate a positive correlation, and light-teal lines indicate a negative correlation.

We explored the interaction between the trans-acting DE-lncRNAs and DE-mRNAs, focusing on those displaying strong correlations. Our analysis identified trans-acting lncRNAs (Supplementary Table S6) associated with key markers of cell proliferation (*CCNA2* and *CDK4*) and myoblast differentiation markers (*MYOG*, *MYF5,* and *MYF6*). A total of 51 trans-acting lncRNAs, including *ENSAPLT00020014663*, *ENSAPLT00020012069*, *ENSAPLT00020002101*, and *ENSAPLT00020016894*, were selected, and 56 nodes and 175 connections were obtained. A network of trans-acting DE-lncRNAs and DE-mRNAs was visualized (Figure 4). This network highlighted both positive and negative correlations in 92 and 83 interactions, respectively.

### 2.6. Expression Profiling and Differential Expression of miRNAs

To investigate the miRNA expression profiles in duck myogenesis, we performed high-throughput miRNA sequencing. The miRNA library for the GM group (GM1–GM3) contained 37,721,143 sequence reads (ranging from 12,103,140 to 12,839,480), whereas the DM group (DM1–DM3) contained 36,304,340 sequence reads (ranging from 11,490,694 to 12,706,500). Subsequent quality filtering yielded 31,607,697 and 29,263,710 clean reads obtained for GM and DM, respectively (Supplementary Table S7). Pearson’s Correlation and a Principal Component Analysis were used to assess the relationship between samples. Our analysis indicated a strong correlation among the GM samples and differences between the GM and DM groups, reflecting distinct miRNA expression patterns in myoblasts and myotubes (Supplementary Figure S2). 

**Figure 5:**
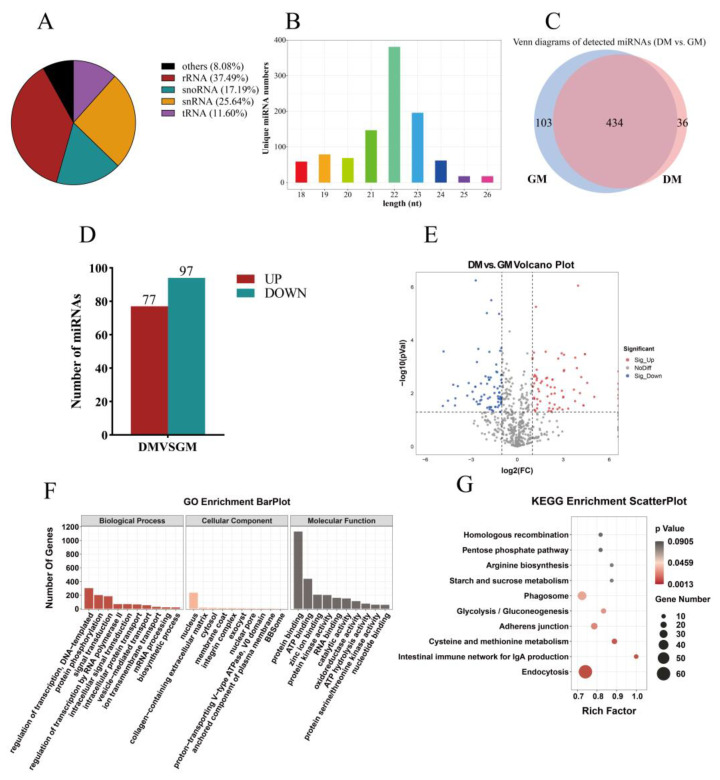
Expression profiling and differential expression of miRNAs in duck myogenesis. (**A**) The type and proportion of detected small RNAs. (**B**) Length distribution of miRNA reads. (**C**) Venn diagrams of detected miRNAs between DM and GM. (**D**) The number of DE-miRNAs in DM and GM. (**E**) Volcano plot for miRNA expression. The vertical dotted lines indicate |log2FC| = 1, and the horizontal dotted lines indicate *p* value = 0.05. (**F**) Top GO terms of DE-miRNAs. (**G**) Top 10 KEGG pathways of DE-miRNAs. nt: nucleotides.

After removing other small ncRNAs, such as rRNA and tRNA (Figure 5A), a total of 1029 miRNAs were obtained, including 572 known miRNAs and 457 novel miRNAs (Supplementary Table S2). The sequence length of these miRNAs ranged from 21 to 23 nt, which was in accordance with the characteristics of miRNAs and proved the reliability of the data (Figure 5B). An expression analysis of these miRNAs showed that 434 miRNAs were expressed in both the GM and DM, whereas 103 miRNAs were only expressed in the GM, and 36 miRNAs were only expressed in the DM (Figure 5C). 

After a differential expression analysis, we identified 174 DE-miRNAs, including 77 DE-miRNAs that were up-regulated and 97 DE-miRNAs that were down-regulated. The volcano plots illustrate the distribution of DE-miRNAs (Figure 5D,E). A heatmap of the DE-miRNAs showed the difference between myoblasts and myotubes (Figure S1D).

To further explore the function of DE-miRNAs, the target mRNAs of DE-miRNAs were enriched and analyzed using the GO and KEGG methods. The GO results showed that the target genes of the DE-miRNAs were significantly enriched in 90 GO terms and mostly enriched in the biological process of the regulation of transcription, DNA-templated, protein phosphorylation, signal transduction, the regulation of transcription via RNA polymerase II, and intracellular signal transduction (Figure 5F and Supplementary Table S8). The KEGG pathway analyses showed that the target genes of the DE-miRNAs were mostly enriched in endocytosis, the intestinal immune network for IgA production, cysteine and methionine metabolism, adherens junction, glycolysis/gluconeogenesis, and phagosome (Figure 5G and Supplementary Table S8).

### 2.7. Construction of ceRNA Interaction Regulatory Network 

**Figure 6:**
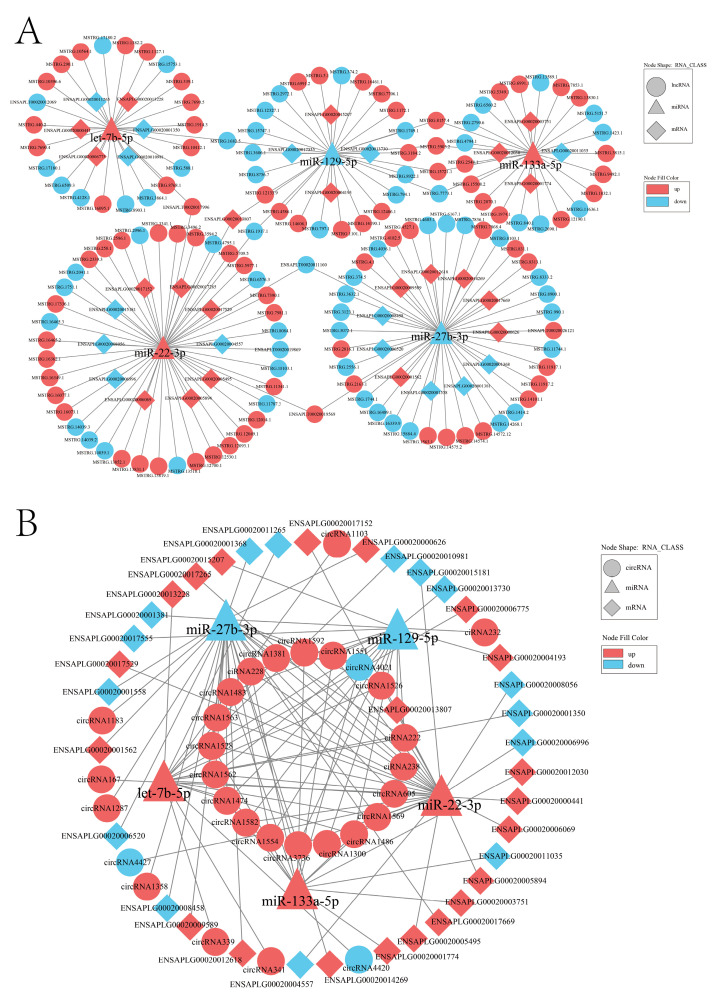
ceRNA co-regulatory network of DE-mRNAs, DE-lncRNAs, DE-circRNAs, and DE-miRNAs. (**A**) Co-regulatory network of lncRNA-miRNA-mRNA. (**B**) Co-regulatory network of circRNA-miRNA-mRNA. The triangle represents miRNAs. Rhombus represents mRNAs. The circle represents lncRNAs or circRNAs. Up-regulation is shown in red and down-regulation is shown in blue.

To explore the regulatory crosstalk between ncRNAs and mRNAs, we constructed a ceRNA interaction network (Supplementary Table S9). This network incorporated interactions between differentially expressed miRNAs and their targets in the context of lncRNAs and circRNAs. Notably, we focused on five miRNAs (miR-129-5p, miR-133a-5p, miR-22-3p, miR-27b-3p, and let-7b-5p) involved in skeletal muscle development. We identified 1275 lncRNA-miRNA-mRNA interaction pairs (Figure 6A) and 588 circRNA-miRNA-mRNA interaction pairs (Figure 6B). Moreover, we pinpointed potential core lncRNAs and circRNAs in regulatory networks, including lncRNA-miRNA-mRNA interaction network, *ENSAPLT00020011160*, *ENSAPLT00020017996*, *ENSAPLT00020019569*, *MSTRG.15721.1*, *MSTRG.1682.5*, *MSTRG.1917.1*, *MSTRG.5903.2*, *MSTRG.7773.1,* and *MSTRG.8157.4*, which were predicted as core DE-lncRNAs, whereas in the circRNA-miRNA-mRNA interaction network, 20 DE-circRNAs, such as *circRNA1562*, *circRNA1474*, *circRNA1483*, *circRNA1486*, *circRNA1528*, *circRNA1569*, *circRNA3736,* and *ciRNA222*, were predicted as molecular sponges of several skeletal muscle development-related miRNAs. These networks offer a comprehensive view of the interactions between miRNAs, lncRNAs, circRNAs, and mRNAs.

**Figure 7:**
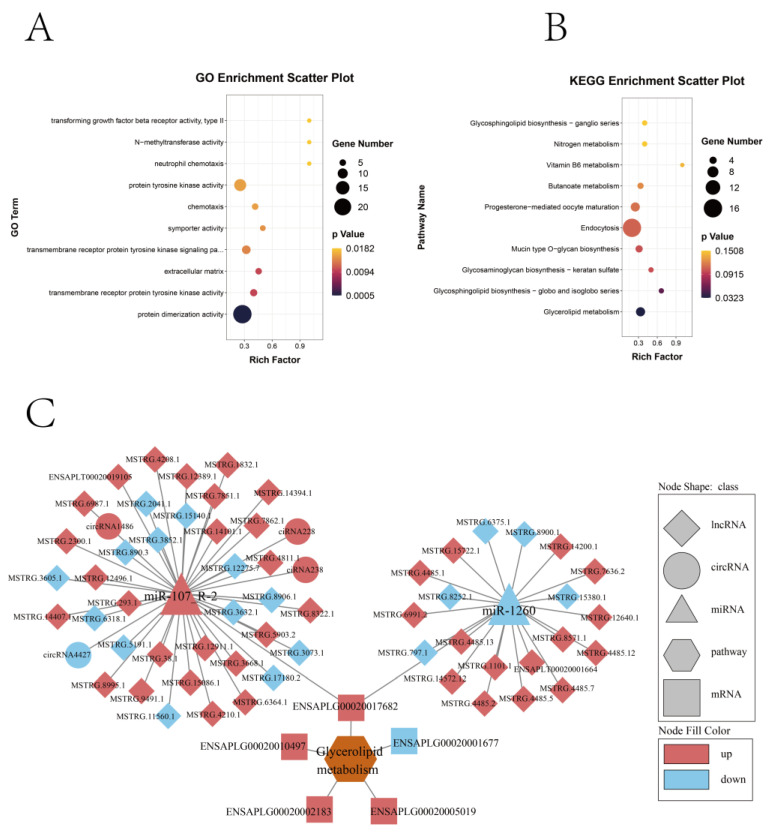
Functional enrichment analysis of mRNAs involved in the ceRNA network and the candidate ceRNA co-regulation network. (**A**) Top 10 GO terms of DE-mRNAs involved in the ceRNA network. (**B**) Top 10 KEGG pathways of DE-mRNAs involved in the ceRNA network. (**C**) LncRNA/circRNA-miRNA-mRNA pathway regulatory network. The triangle represents miRNAs. Rhombus represents lncRNAs. The square represents mRNAs. The circle represents circRNAs. The hexagon represents a pathway. Red nodes indicate up-regulation, and blue nodes indicate down-regulation.

Based on DE-mRNAs involved in the regulatory network of lncRNA/circRNA-miRNA-mRNA, an enrichment analysis of GO and KEGG was carried out (Figure 7A,B and Supplementary Table S10). The results showed that the genes related to miRNA, lncRNA, and circRNA were significantly enriched in glycerolipid metabolism. Five DE-mRNAs, including *ENSAPLG00020001677*, *ENSAPLG00020002183*, *ENSAPLG00020005019*, *ENSAPLG00020010497*, and *ENSAPLG00020017682* (enriched in the pathway of glycerolipid metabolism), were considered as potential target genes for myoblast differentiation. To further explore the ceRNA network involved in myoblast differentiation, a ceRNA interaction regulatory network was constructed for the above five mRNAs. Finally, we established a ceRNA interaction network (Figure 7C), which included 5 mRNAs, 2 miRNAs, 57 lncRNAs, and 4 circRNAs. The results showed that miR-1260 and miR-107_R-2 were involved in the regulation of the glycerolipid metabolism pathway, which might be one of the key candidate factors for regulating myogenesis.

### 2.8. qRT-PCR Validation

**Figure 8:**
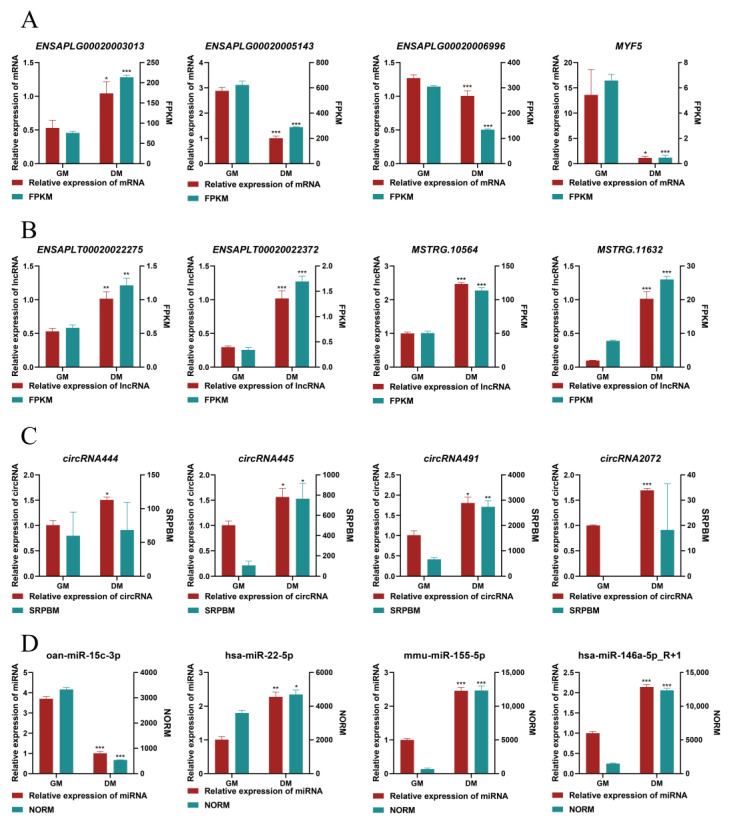
qRT-PCR validation of DE-mRNAs (**A**), DE-circRNAs (**B**) DE-lncRNAs (**C**), and DE-miRNAs (**D**). FPKM: fragment per kilobase of transcript per million mapped reads. SRPBM: spliced reads per billion mapping. NORM: global mean normalizers for miRNA quantification. Data (3 biological replicates) are presented as means ± s.e.m. * *p* < 0.05, ** *p* < 0.01, and *** *p* < 0.001.

To confirm the reproducibility and accuracy of the DE molecules from our RNA-seq data, we performed a qRT-PCR analysis for four randomly selected DE-mRNAs, DE-lncRNAs, DE-circRNAs, and DE-miRNAs (Figure 8). The expression patterns of these molecules in both the GM and DM groups were highly consistent with the results of the RNA-seq. 

## 3. Discussion

Since the 1980s, protein-coding transcripts have been studied extensively, and more recently, non-coding miRNAs have garnered significant attention. However, there is very little published information on the expression patterns and regulatory mechanisms of lncRNAs and circRNAs in duck cell development. Moreover, researchers’ understanding of how these RNAs are regulated by well-established pathways is limited. In this study, we employed an Illumina whole-transcriptome analysis to investigate the expression profiles of mRNAs, lncRNAs, circRNAs, and miRNAs during duck myoblast cell differentiation. Our results revealed noteworthy insights into the molecular landscape of duck cell development.

In this investigation, we identified 1733 DE-mRNAs, 1116 DE-lncRNAs, 54 DE-circRNAs, and 174 DE-miRNAs. Interestingly, the number of DE-mRNAs observed in our study is comparable to those observed in Hanzhong Ma duck skeletal muscle breast tissue [18], suggesting consistency in the gene expression changes across different duck cell types. Nonetheless, variations in the cell type likely explain the differences in the results, as supported by our previous work, which identified a larger number of circRNAs in Shan Ma duck breast muscle tissues at different embryonic stages [1]. Comparative studies, such as the analysis conducted by Chen et al. [19] in Shitou goose, reported a higher number of DE-lncRNAs than we observed in our study. Our results highlight the prevalence of intergenic lncRNAs and exonic circRNAs, which is in line with previous research [20,21]. These findings infer that the numbers of DE-mRNAs, DE-lncRNAs, DE-circRNAs, and DE-miRNAs exhibit considerable diversity across different species, time periods, and cell processes, indicating that the regulatory roles of lncRNAs and circRNAs are contingent upon the specific factors at play in each particular case.

During a biological process, such as in a cell or organism, the gene expression patterns change [22]. During myoblast differentiation, the expression of functional genes, such as *MYOG*, significantly increases, while unrelated genes are repressed [23]. In our comparison of differentiated myoblasts (DM) and proliferating myoblasts (GM), we observed a general up-regulation of mRNAs, lncRNAs, and circRNAs, with fewer miRNAs exhibiting up-regulation. This phenomenon suggests that enhanced differentiation leads to the activation of functional RNAs, while miRNA regulation becomes less pronounced, facilitating the differentiation process and subsequent fusion of myoblasts.

Our study identified DE mRNAs that are crucial for skeletal muscle development, including *MYF5*, *MYOG*, *PAX3*, and *FABP2*. These genes play vital roles in embryonic myogenesis. Notably, *MYF5* and *MYOG* deficiencies can lead to skeletal muscle loss in mice [24,25], emphasizing their significance. In this study, the *FABP2* gene was significantly down-regulated in myoblasts. FABP2 is an intracellular protein, which can regulate important biological processes, such as fatty acid transport and metabolism [26]. *FABP2* has been proven to be DE in the skeletal muscle of white Muscovy ducks with different embryo ages, and the expression level increases with the increase in the embryo age, which may be an important candidate gene for influencing duck growth traits [27]. In recent years, lncRNAs have gained prominence for their roles in muscle growth and atrophy [28]. These functional lncRNAs primarily exert cis-regulation and trans-regulation effects [29]. For example, *lncEDCH1* promotes myoblast proliferation, inhibits differentiation, and reduces intramuscular fat deposition [30]. *LncIRS1* acts as a molecular sponge, regulating the IGF1-PI3K/AKT pathway and controlling muscle atrophy [31]. Our study revealed the significant enrichment of the cis-regulated target genes of DE-lncRNAs in the cell adhesion molecules pathway, which plays a pivotal role in muscle fiber formation and development. Cell adhesion factors are important determinants for myogenesis and skeletal muscle satellite cell activity [32]. Intercellular adhesion molecule-1 (ICAM-1) has been found to play an important role in skeletal muscle cells, which can enhance myogenesis [33]. Trans-acting lncRNAs are known to influence the expression of mRNA genes at a distance from the lncRNA locus. Our analysis identified trans-acting lncRNAs associated with cell proliferation and myoblast differentiation markers, providing insights into their roles in skeletal muscle growth and development [29]. *MUNC* is an ncRNA located upstream of *MYOD* (at the distal regulatory region of *MYOD*). *MUNC* is a trans-acting lncRNA, which is involved in the regulation of many muscle-related genes [34]. LncRNA *PAM* regulates the proliferation and aging of skeletal muscle satellite cells via the trans-regulation of the expressions of *TIMP2* and *VIM* [35]. In our study, we analyzed the interaction between DE-trans-acting lncRNAs and DE-mRNAs, screened out the trans-acting lncRNAs of the cell proliferation and myoblast differentiation markers, and constructed a trans-regulation lncRNA-mRNA interaction regulatory network. The predicted trans-acting lncRNAs found in this study provide new clues for exploring the growth and development of skeletal muscle.

CircRNAs have recently emerged as essential regulators in skeletal muscle development [36]. For instance, *circHIPK3* regulates the expression of *MEF2C* by acting as a miR-30a-3p sponge, thereby promoting the proliferation and differentiation of chicken myoblasts [37]. Research has shown that *circMYBPC1* functions as a miR-23a sponge to enhance muscle differentiation and binds directly to MyHC protein, affecting muscle development [38]. Furthermore, *circ-ZNF609* has an open reading frame with both start and stop codons, enabling it to translate proteins and promote human myoblast proliferation [39]. In our study, we observed that DE-circRNAs significantly enriched the regulation of the actin cytoskeleton signaling pathway. The regulation of the actin cytoskeleton signaling pathway has been found to be significantly enriched in duck breast muscle and leg muscle at different growth stages [40]. In a previous study that conducted a differential expression analysis of circRNAs in the skeletal muscle of juvenile and adult largemouth bass, it was found that the parent gene is mainly enriched in the actin cytoskeleton regulation signaling pathway [41]. At the same time, the parental genes of circRNAs with coding potential in the expression profile of mouse C2C12 myoblasts were also enriched into the regulation of the actin cytoskeleton signaling pathway [42]. This pathway has been consistently implicated in skeletal muscle development in various species, further highlighting its importance.

Regarding the regulatory mechanism of ncRNAs, the ceRNA hypothesis was first put forward in 2011 and was widely accepted and used to explain various genetic mechanisms [43]. It is important to reveal the potential molecular mechanism of duck skeletal muscle growth and development; however, the regulatory mechanism of ceRNA is limited in the study of duck skeletal muscle. MiRNAs can directly target mRNAs to regulate gene expression and often interact with mRNAs and ncRNAs via the ceRNA regulation mechanism [44]. For instance, in skeletal muscle regeneration and myogenesis, miR-200c-5p regulates the migration and differentiation of muscle cells by targeting *Adamts5* [11]. Based on the results of the miRNA-seq, we identified many DE-miRNAs, including miR-129-5p [45], miR-133a-5p [46], miR-22-3p [47], miR-27b-3p [48] and let-7b-5p [49]. Importantly, we revealed some potential ceRNA regulatory networks before and after the differentiation of duck embryo myoblasts based on the interaction regulatory networks of DE-miRNA with DE-mRNA, DE-miRNA with DE-lncRNA, and DE-miRNA with DE-circRNA. *MSTRG.8157.4*, *MSTRG.5093.2*, *MSTRG.15721.1*, *MSTRG.7773.1*, *MSTRG.1682.5*, *MSTRG.1917.1*, *ENSAPLT00020011160*, *ENSAPLT00020019569*, *ENSAPLT00020017996*, 9 DE-lncRNAs, and 20 DE-circRNAs, including *circ4021*, *circ1551*, and *circ1562*, can simultaneously act as molecular sponges for the five miRNAs. A functional enrichment analysis was carried out based on all DE-mRNAs involved in these ceRNA networks. Interestingly, a KEGG enrichment analysis showed that these DE genes were significantly enriched in the glycerolipid metabolism signaling pathway. The regulation of glycerolipid biosynthesis is important for cell lipid storage and cell membrane homeostasis [50]. The further construction of the lncRNA/circRNA-miRNA-mRNA-pathway regulation network showed that miR-1260 and miR-107_R-2 were involved in the regulation of the glycerolipid metabolism pathway. It was found that miR-1260 and miR-107 were closely related to the expression of cytochrome P450 (CYP) enzymes, including CYP3A4, CYP3A5, and CYP3A43 [51]. MiR-107 is highly expressed in the skeletal muscle of Qinchuan cattle, and *circFGFR4* can promote myoblast differentiation by binding with miR-107 [52]. In this study, we found that miR-1260 and miR-107_R-2 were highly expressed in the primary myoblasts of duck embryos. miR-1260 was significantly down-regulated, and miR-107_R-2 was significantly up-regulated in the primary myoblasts of duck embryo. This indicates that miR-1260 and miR-107_R-2 may be involved in the regulation of the skeletal muscle–glycerolipid metabolism pathway, thus affecting the differentiation of skeletal muscle and becoming potential candidate miRNAs for regulating the growth and development of skeletal muscle. These ceRNA networks and the trans-acting lncRNA-mRNA interaction constructed in this study can be used as candidate regulatory networks for the differentiation of primary myoblasts from duck embryos. 

In conclusion, this study offers a comprehensive characterization of mRNA and ncRNA (lncRNA-circRNA-miRNA) expression profiles during duck leg myogenesis, providing valuable insights into the molecular mechanisms governing healthy duck muscle development. The identified ceRNA networks and lncRNA-mRNA interactions serve as candidate regulatory networks for further exploration in the context of duck skeletal muscle differentiation and growth. These findings lay the groundwork for future research in this field.

## 4. Materials and Methods

### 4.1. Isolation, Culture, and Differentiation of Primary Myoblasts from Duck Embryos

Duck embryo primary myoblasts were isolated from leg muscles of embryonic day 13 (E13) and grown as previously described [53]. Briefly, leg muscle tissue was dissected from skin and bone and homogenized in a centrifuge tube. A single-cell suspension was obtained by digesting with 0.25% Trypsin-EDTA (Gibco, Waltham, MA, USA) at 37 °C for 20 min, followed by termination of digestion with FBS (Gibco, Waltham, MA, USA). Subsequently, the single-cell suspension was filtered, and non-adherent myoblasts were collected via centrifugation at 1500 rpm for 5 min. To remove fibroblasts, differential adhesion was performed three times, retaining myoblasts that did not adhere to the cell culture plate. Myoblasts were cultured into 60 mm cell culture dishes in a growth medium comprising 10% FBS (Gibco, Waltham, MA, USA) and Dulbecco’s Modified Eagle Medium (DMEM), purchased from Gibco (Waltham, MA, USA). When the cell density reached 90%, proliferating myoblasts (GM) were harvested. Upon reaching 90% confluence, the proliferating myoblasts were induced to differentiate in DMEM supplemented with 2% horse serum (Gibco, Waltham, MA, USA) and 0.3% penicillin/streptomycin, and the cells in differentiation medium (DM) were collected on day 4 of differentiation (myotubes). All cells were cultured in a cell incubator with 5% CO_2_ and a humidified atmosphere at 37 °C.

### 4.2. Immunofluorescence Analysis

For immunofluorescence analysis, proliferating myoblasts and myotubes at day 4 of differentiation were fixed in 4% paraformaldehyde and stained in PBS containing 10% goat serum. Anti-desmin primary antibody (rabbit monoclonal, bs-1026R, Bioss, Woburn, MA, USA) was used at a dilution of 1:150, and MyHC primary antibody (MF20, DSHB, Iowa City, IA, USA) was used at a dilution of 3 µg/mL. A secondary fluorochrome-conjugated antibody (M21012M, Abmart, Shanghai, China) was used at a dilution of 1:300, and a secondary fluorochrome-conjugated antibody (FITC, A0568, Beyotime, Shanghai, China) was used at a dilution of 1:500. Images were randomly captured with a fluorescence microscope (TS2R-FL; Nikon, Tokyo, Japan).

### 4.3. RNA Isolation, Library Preparation, and Sequencing

Proliferating myoblasts and myotubes at day 4 of differentiation were collected from three biological replicates, totaling six samples. Total RNA was extracted from these samples using TRIzol (Thermo Fisher, Waltham, MA, USA) and stored at −80 °C. The samples were sent to LC-BIO (Hangzhou, China) for whole-transcriptome RNA sequencing. The purity and concentration of the total RNA were analyzed using the RNA 6000 Nano Lab Chip Kit (Agilent, San Diego, CA, USA, 5067-1511) and Bioanalyzer 2100 (Agilent), ensuring an RIN number >7.0.

For mRNA, lncRNA, and circRNA sequencing, ribosomal RNA (rRNA) was removed from 5 µg of total RNA using the Ribo Zero Gold rRNA removal kit (Illumina, San Diego, CA, USA). The average insertion length of the final cDNA library was 300 bp (±50 bp), and libraries were sequenced with 2 × 150 bp (pair-end reads) on the Illumina Nova Seq™ 6000 (LC-BIO, Hangzhou, China).

For miRNA library construction, TruSeq Small RNA Sample Prep Kits (Illumina, San Diego, CA, USA) were used according to the manufacturer’s instructions. In short, 3 ug of the total RNA was added to 3′ and 5′ adapter-ligated RNA. Subsequently, reverse transcription PCR was performed to amplify the RNA with the ligated adapter, and then gel purification was carried out. Finally, the cDNA product was sequenced with 1 × 50 bp single-end read sequencing on the Illumina HiSeq 2500 (LC-BIO, Hangzhou, China).

### 4.4. Identification of lncRNA, circRNA, and miRNA

High-throughput-sequencing reads were processed to remove the sequences containing adapter contamination, low-quality bases, and undetermined bases using Cutadapt (v1.9) [54]. FastQC (v0.10.1, Nanjing Agricultural University, Nanjing, China) was used to assess sequence quality, and reads were mapped to the Mallard duck reference genome (ASM874695v1) using Bowtie (v2.5.1) [55] and Hisat2 (v2.2.1, Iowa State University, Ames, IA, USA) [56].

*Identification of lncRNA*: StringTie (v2.1.6, Johns Hopkins University, Baltimore, MD, USA) [57] was used to identify novel transcripts compared to the reference genome. Gffcompare (v0.9.8, Johns Hopkins University, Baltimore, MD, USA) was used to filter known mRNA, lncRNA, and transcripts shorter than 200 nt. We implemented a systematic approach to define lncRNA transcripts. Specifically, we classified transcripts with class codes ‘i, j, o, u, and x’ as lncRNAs. These class codes signify distinct features that are indicative of lncRNA characteristics. Let us delve into these classifications:

(i) Class ‘i’ refers to a transcribed fragment, which could be either in the sense or anti-sense orientation, that is entirely contained within a reference intron. This delineates the lncRNAs associated with intronic regions.

(j) Class ‘j’ pertains to transcripts that exhibit at least one splicing junction shared with the reference transcript. This classification highlights lncRNAs with splicing features in common with known transcripts.

(u) Class ‘u’ designates a lncRNA as an intergenic transcript of unknown function. This category encompasses lncRNAs originating from intergenic regions, often with yet undetermined functions.

(o) Class ‘o’ is applied when an ordinary exon of a predicted lncRNA partially overlaps with a reference transcript. This classification recognizes lncRNAs that may share exonic sequences with other transcripts. CPC (v0.9-r2, Tsinghua University, Beijing, China) [58] and CNCI (v2.0, Chinese Academy of Sciences, Beijing, China) [59] software were used to predict novel lncRNA transcripts with coding potential. All transcripts with CPC score < 0.5 and CNCI score < 0 were considered novel lncRNAs.

Identification of circRNA: From the aligned BAM files, circRNA sequences were identified using CircExplorer (v2.2.6) [60,61] and CIRI (v2.0.2) [62]. The reverse splicing sequences were identified in the unmapped sequence using TopHat-Fusion (v2.0.10) [63], a bioinformatic tool used for the detection of gene fusions.

Identification of miRNA: ACGT 101-miR (LC Sciences, Houston, TX, USA) was used to remove adapter dimer, garbage, low complexity, and common RNA families (rRNA, tRNA, snRNA, and snoRNA), followed by mapping unique sequences (with a length of 18~26 nucleotides) to specific species precursors in miRBase (Release 22.0, www.mirbase.org/, accessed on 1 August 2022.) [64] using BLAST search to identify known and novel miRNAs. Length variations at both the 3′ and 5′ ends, as well as mismatches within non-identical sequences, are considered during the comparison process. We employed this approach to identify mature miRNA hairpin arms with distinctive sequences, which were mapped to specific species, thereby classifying them as known miRNAs. Conversely, in instances where the sequence of a mature miRNA exhibited annotations that corresponded to the opposite arm of a species-specific precursor hairpin, we identified them as novel 5p- or 3p-derived miRNA candidates.

### 4.5. Analysis of Differentially Expressed (DE) Genes

The FPKM (fragment per kilobase of transcript per million mapped reads) value [65] was computed using StringTie (v2.1.6, Johns Hopkins University, Baltimore, MD, USA) and R ballgown package (v2.18.0) [66] to estimate the expression levels of mRNAs and lncRNAs. For circRNAs, we used the SRPBM (the Spliced Reads Per Billion Mapping) method to determine their normalized expression. To identify and quantify small RNAs, specifically miRNAs, in our samples, we relied on the ACGT101-miR tool (LC Sciences, Houston, TX, USA). This tool allowed us to pinpoint miRNAs and determine their expression levels. To ensure data consistency, we applied a previously described normalization procedure [67], resulting in the ‘norm’ value, which serves as a robust indicator of the miRNA expression. DE-genes, including mRNAs, circRNAs, lncRNAs, and miRNAs, were screened under the conditions of either a log2 (fold change) greater than 1 or less than −1, along with an adjusted *p*-value below 0.05.

### 4.6. Screening Potential Target Genes of Differentially Expressed circRNA/lncRNA/miRNA and Functional Enrichment Analysis of GO and KEGG

The distinct functions and modes of action of lncRNA, circRNA, and miRNA necessitated the use of distinct screening methods in our RNA sequencing and analysis. In the case of DE-lncRNAs, we employed Python scripts to screen for cis-acting lncRNAs. Specifically, we identified lncRNAs with a positional relationship to mRNAs within a range of 100 kb both upstream and downstream of the chromosomes. These lncRNAs were considered as potential cis-acting regulators, and the target mRNAs were obtained through the prediction of interactions between these cis-acting lncRNAs and DE-mRNAs. For the DE-circRNAs, we considered their host genes as potential target genes. In the case of the DE-miRNAs, we utilized two databases, TargetScan (v5.0) [68] and miRanda (v3.3a) [69], for the prediction of target genes with significant differences. We refined our predictions by applying stringent scoring criteria, including a TargetScan_score threshold of ≥50 and a miRanda_Energy below −10, ensuring that only the most relevant target genes were considered as the final target genes of differential miRNA.

Subsequently, DE-mRNAs, DE cis-acting lncRNAs, DE-circRNAs, and DE-miRNAs underwent an enrichment analysis using GO and KEGG. The top GO terms and signaling pathways were visually represented using a bubble chart, with significance determined using the enriched *p*-values. This visualization was generated through the R-packet, ggplot2.

### 4.7. Analysis of the Interaction between Trans-Acting lncRNA and mRNA

Trans-acting lncRNAs play pivotal roles in regulating gene expression across different chromosomes. The identification of target mRNAs for trans-acting lncRNAs is based on the determination of the free energy required to form a stable secondary structure between the lncRNA and the mRNA sequence. A lower free energy requirement indicates a greater potential for an interaction between these two sequences. To predict the target mRNAs of the trans-acting lncRNA, we employed the RIsearch (v1.1) software [70], which utilizes this free energy criterion to make precise predictions. Specifically, we assessed the likelihood of an interaction by considering the energetic stability of the secondary structure formed between the lncRNA and its target mRNA sequences. Our focus extended to trans-acting lncRNAs that interacted with key markers of cell proliferation, such as *CCNA2* and *CDK4*, as well as markers of myoblast differentiation, including *MYOG*, *MYF5*, and *MYF6*. To ensure the robustness of these interactions, we applied a stringent criterion, requiring an absolute value of the Pearson’s correlation coefficient greater than 0.8. This selection process ensured that only highly correlated trans-acting lncRNAs were considered. To provide a comprehensive visualization of these intricate interactions, we utilized the Cytoscape (v3.8.2, Institute for Systems Biology, Seattle, Washington, DC, USA) software [71].

### 4.8. Construction of lncRNA/circRNA-miRNA-mRNA ceRNA Network

To explore the interaction among DE-mRNA, DE-lncRNA, DE-circRNAs, and miRNAs with statistical significance *p* < 0.05, we constructed a regulatory network based on the ceRNA theory, which links lncRNA/circRNA-miRNA-mRNA interactions. The approach began with the prediction of miRNA-mRNA, miRNA-lncRNA, and miRNA-circRNA pairs using TargetScan (v5.0) (with a score threshold of ≥50) and miRanda (v3.3a) (with an energy threshold of <−10) software. These stringent criteria ensured the selection of high-confidence interactions that were likely to be biologically relevant.

From this pool of interactions, we focused on five miRNAs known to be involved in skeletal muscle development. For these miRNAs, we imposed even stricter criteria, requiring a TargetScan_score of ≥90 and a miRanda_Energy of <−15 for both miRNA-mRNA and miRNA-lncRNA interactions. Next, we constructed the miRNA-circRNA ceRNA network under the previously established conditions (TargetScan score ≥ 50 and miRanda_Energy < −10), further extending our understanding of regulatory interactions. Following this, we conducted GO and KEGG analyses to identify DE-mRNAs involved in the ceRNA regulatory network. This allowed us to elucidate the biological pathways and processes affected by these interactions. To provide a visual representation of this complex network, we employed Cytoscape (v3.8.2, Institute for Systems Biology, Seattle, DC, USA) [71].

### 4.9. qRT-PCR

To validate the expression levels of DE-mRNAs, DE-lncRNAs, DE-circRNAs, and DE-miRNAs, we employed qRT-PCR. Specifically, we randomly selected four genes from each of the aforementioned RNA categories that exhibited significant DE expressions. For the qRT-PCR experiments, we utilized six RNA samples, including myoblasts (*n* = 3) and myotubes (*n* = 3). The primers used for mRNAs and lncRNAs were designed through the primer-BLAST tool (https://blast.ncbi.nlm.nih.gov/Blast.cgi?PROGRAM=blastn&PAGE_TYPE=BlastSearch&LINK_LOC=blasthome, accessed on 13 April 2023.) available in the NCBI database. As for circRNAs, primer design was carried out using Oligo7.0 software (Thermo Scientific, Waltham, MA, USA). The information of all primers used in the current study is provided in Supplementary Table S11.

To detect miRNA, we employed a specific miRNA bulge-loop qRT-PCR primer set, designed by Ribobio (Guangzhou, China). The quantification of miRNAs was achieved through qPCR using the Revertra Ace qPCR RT Kit (No.FSQ-101, Toyobo, Osaka, Japan).

To prepare the RNA for qPCR analysis, reverse transcription into cDNA was performed using HIScript III All-in-one RT Supermix Perfect for qPCR (R333-01, Vazyme, Nanjing, China).

The qPCR reactions were carried out using the 2 × T5 fast qPCR mix (SYBR Green I) (tse202, TSINGKE, Beijing, China) within a 20 µL reaction system. The components included 3 µL cDNA, 0.2 µL of 50 × ROX Reference Dye II, 0.4 µL each of upstream and downstream primers, 5 µL SYBR Green I, and 11 µL of DEPC water. The qPCR reaction was carried out under the following steps: an initial temperature at 95 °C for 1 min, followed by 40 cycles of denaturation at 95 °C for 10 s, annealing at 60 °C for 5 s, and extension at 72 °C for 15 s. A final extension step was conducted at 72 °C for 7 min. *GAPDH* was utilized as the internal reference for mRNA, lncRNA, and circRNA analyses, while *U6* served as the internal reference for miRNA analysis. The 2^−ΔΔCT^ method [72] was used to calculate the relative expression level.

The qPCR results are presented as means ± s.e.m. For multiple comparison analysis on relative expression of muscle markers, groups were compared with a one-way ANOVA test followed by a Duncan test using SPSS 26.0. The different letters between the two groups represent significant differences (*p* < 0.05). For two-group comparison on the validation of differentially expressed RNAs, the results were subjected to statistical analysis using the two-tailed Student’s *t*-test. The level of significance was presented as * *p* < 0.05, ** *p* < 0.01, and *** *p* < 0.001.

## Figures and Tables

**Table 1:** The numbers of DE-mRNAs, DE-lncRNAs, and DE-circRNAs.

Terms	mRNAs		lncRNAs		circRNAs	
	up	down	up	down	up	down
DM vs. GM	1065	668	653	463	44	10

Note: Up, up-regulated; Down, down-regulated.

## Data Availability

This study’s raw sequencing data and processing files were deposited into the China National Center for Bioinformation (https://www.cncb.ac.cn/, accessed on 31 July 2023.) with the accession number of ‘CRA012056’.

## Supplementary Materials

The following supporting information can be downloaded at https://www.mdpi.com/article/10.3390/ijms242216387/s1.
