# Supplementary material for: Whole-Transcriptome RNA Sequencing Uncovers the Global Expression Changes and RNA Regulatory Networks in Duck Embryonic Myogenesis
Source: Int J Mol Sci. 2023 Nov 16;24(22):16387. doi: 10.3390/ijms242216387 (PMC10671564; doi:10.3390/ijms242216387)
Supplement: Supplementary file 1 [file ijms-24-16387-s001.zip › Supplementary Table S1. Summary of the RNA-seq data.pdf]

Supplementary Table S1. Summary of the RNA-seq data

Sample	DM1	DM2	DM3	GM1	GM2	GM3
Raw reads	87303056	85278832	82812096	86647062	74888572	87590522
Valid reads	68004646	64394120	64469736	68085998	61505334	67148268
Valid ratio, %	77.89	75.51	77.85	78.58	82.13	76.66
Mapped reads	61403154	57915458	58016166	60780743	55597673	60501279
Mapped ratio, %	90.29	89.94	89.99	89.27	90.39	90.10
Unique mapped reads	58201918	54557090	54930709	57272551	53097607	57276269
Unique ratio, %	85.59	84.72	85.20	84.12	86.33	85.30
Q30, %	97.74	97.64	97.66	97.58	97.95	97.76
GC content, %	47.50	48.50	47.50	47.50	45.50	47.50
