# Supplementary material for: Whole-Transcriptome RNA Sequencing Uncovers the Global Expression Changes and RNA Regulatory Networks in Duck Embryonic Myogenesis
Source: Int J Mol Sci. 2023 Nov 16;24(22):16387. doi: 10.3390/ijms242216387 (PMC10671564; doi:10.3390/ijms242216387)
Supplement: Supplementary file 1 [file ijms-24-16387-s001.zip › Supplementary Table S11. The primers used for qRT-qPCR.pdf]

Supplementary Table S11. The primers used for qRT-qPCR

Type	Gene name	Primer sequences (5'-3')
mRNA	ENSAPLG00020003013	F: CCGGACAACTTGCACAAAGG
		R: TGGTGTGCTTAGCCATCTCC
	ENSAPLG00020005143	F: ATATAAAAGGGGCGCGGTAGG
		R: TGGCTGTTTGAGAAGGGCAT
	ENSAPLG00020006996	F: CAAAGATGCGCTGGATGAGC
		R: GGTAGCATCTTCAGGCCTCC
	MYF5	F: GGCGTTTGAGACCCTGAAGA
		R: TGTCCCGGCAGGTGATAGTA
	MYOG	F: CCGGACCAGAGGTTTTACGA
		R: CAAAGCCACCCTGCTTTCG
	MYOD	F: CGACGGCATGATGGAGTACA
		R: TGAGAGGCAATCAAGGCTGG
lncRNA	ENSAPLT00020022372	F: CTTGATGCATTTGGATGGCCC
		R: AAGGTATGCTGCCATTCCCA
	ENSAPLT00020022275	F: CCTGGCTCATGCCTATCGAG
		R: AGAATCCACCGTAGGGGTCA
	MSTRG.11632	F: CGTATGTTTTCAGCACTCCCTG
		R: AGCACATTAAGATGCAGGCAAA
	MSTRG.10564	F: ACGGATGCAAGTCTTGGGTT
		R: GTGGGGGTGTGGGGTTAAAT
circRNA	circRNA444	F: TCATTGTGCTTGTCCACAGC
		R: AGAGGTTGGCCAGCATGA
	circRNA445	F: TGGTCATTGTGCTTGTCCACAG
		R: TGACAGTGTTCCATTCAGTGGC
	circRNA491	F: CCCCATGAAAAAGACCAGGTTGAA
		R: CTGCTGGCTGAAACTTCCAACT
	circRNA2072	F: TGGGAGATTACTGAAGGATGGAC
		R: CAATATGGACAGGCAGGAAGG
control	GAPDH	F: GGTAGTGAAGGCTGCTGCTGATG
		R: CCACCACACGGTTGCTGTATCC
