# Supplementary material for: Whole-Transcriptome RNA Sequencing Uncovers the Global Expression Changes and RNA Regulatory Networks in Duck Embryonic Myogenesis
Source: Int J Mol Sci. 2023 Nov 16;24(22):16387. doi: 10.3390/ijms242216387 (PMC10671564; doi:10.3390/ijms242216387)
Supplement: Supplementary file 1 [file ijms-24-16387-s001.zip › Supplementary Table S7. Summary of the miRNA-seq data.pdf]

Supplementary Table S7. Summary of the miRNA-seq data

Sample	DM1	DM2	DM3	GM1	GM2	GM3
Raw reads	12107146	12706500	11490694	12839480	12778523	12103140
Clean reads	10276160	9656231	9331319	10572470	10487255	10547972
Clean ratio, %	84.88	75.99	81.21	82.34	82.07	87.15
valid reads	1617964	1658511	1343026	2108257	2068623	2013243
Valid ratio, %	13.36	13.05	11.69	16.42	16.19	16.63
