# Supplementary figures and images for: Whole-Transcriptome RNA Sequencing Uncovers the Global Expression Changes and RNA Regulatory Networks in Duck Embryonic Myogenesis
Source: Int J Mol Sci. 2023 Nov 16;24(22):16387. doi: 10.3390/ijms242216387 (PMC10671564; doi:10.3390/ijms242216387)

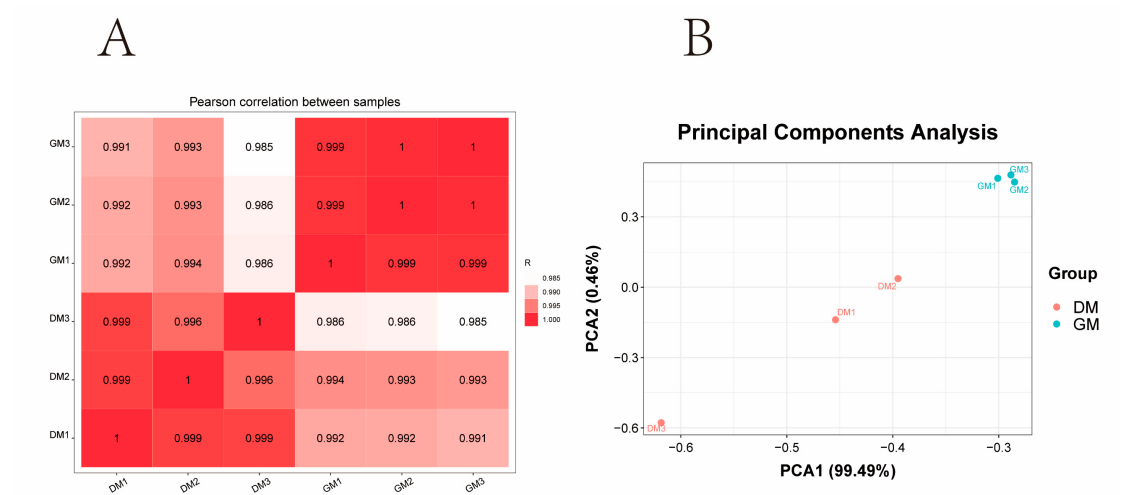

**Figure S2.** Pearson's Correlation (A) and Principal Component Analysis (B) of the miRNA-seq data

Supplement: Supplementary file 1 [file ijms-24-16387-s001.zip › Supplementary Figure S2. Pearsoní»s Correlation and Principal Component Analysis of the miRNA-seq data.pdf]
