# Supplementary material for: Whole-Transcriptome RNA Sequencing Uncovers the Global Expression Changes and RNA Regulatory Networks in Duck Embryonic Myogenesis
Source: Int J Mol Sci. 2023 Nov 16;24(22):16387. doi: 10.3390/ijms242216387 (PMC10671564; doi:10.3390/ijms242216387)
Supplement: Supplementary file 1 [file Supplementary Figure S1. Heatmaps of DE-mRNAs, DE-lncRNAs, DE-circRNAs, DE-miRNAs.pdf › Supplementary Figure S1. Heatmaps of DE-mRNAs, DE-lncRNAs, DE-circRNAs, DE-miRNAs.pdf]

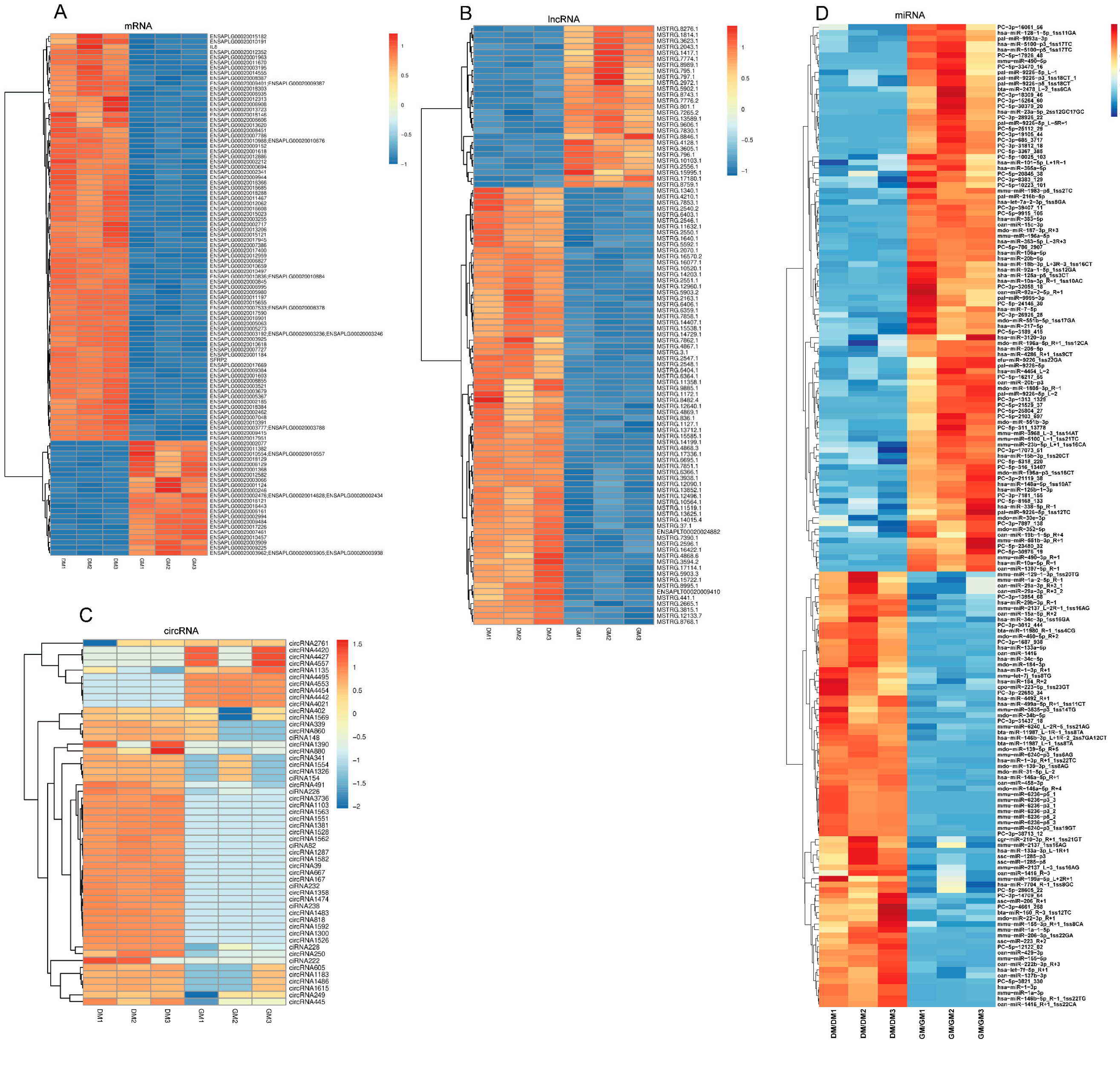

Figure S1. Heatmaps of DE-mRNAs (A), DE-lncRNAs (B), DE-circRNAs (C), DE-miRNAs (D).
